# Exercise training suppresses scavenger receptor CD36 expression in kupffer cells of nonalcoholic steatohepatitis model mice

**DOI:** 10.14814/phy2.13902

**Published:** 2018-12-04

**Authors:** Noriaki Kawanishi, Tsubasa Mizokami, Koichi Yada, Katsuhiko Suzuki

**Affiliations:** ^1^ Graduate School of Sport Sciences Waseda University Tokorozawa Saitama Japan; ^2^ Faculty of Advanced Engineering Chiba Institute of Technology Narashino Chiba Japan; ^3^ Research Fellow of the Japan Society for the Promotion of Sciences Chiyoda‐ku, Tokyo Japan; ^4^ Faculty of Sport Sciences Waseda University Tokorozawa Saitama Japan

**Keywords:** CD36, exercise training, macrophage, PPAR‐γ

## Abstract

Although nonalcoholic steatohepatitis (NASH) is an important component of the metabolic syndrome, scavenger receptor CD36 also modulates NASH development. This study aimed to clarify whether exercise training suppresses CD36 expression in a mouse model of NASH. Male C57BL/6 mice were divided into four groups: normal diet (ND) sedentary, ND exercise, high‐fat diet and high‐fructose water (HFF) sedentary, and HFF exercise groups. The exercise groups were trained on a motorized treadmill at running speeds of 15–20 m/min for 60 min/day, 5 times/week for 16 weeks. CD36 cell surface expression of hepatic resident macrophages, peroxisome proliferator‐activated receptor (PPAR)‐γ protein, and mRNA levels in the liver were increased in HFF sedentary mice but were attenuated in HFF exercise mice. Hepatic resident macrophages were significantly lower in HFF exercise mice than in HFF sedentary mice. Our findings indicated that exercise training reduced macrophage quantity in the liver, and downregulated CD36 and PPAR‐γ expression in liver and macrophages.

## Introduction

Nonalcoholic steatohepatitis (NASH) is an important component of the metabolic syndrome (Browning and Horton 2004). The innate immune system plays a key role in the development of NASH (Maher et al. 2008). Kupffer cells are resident macrophages in the liver and they produce various pro‐inflammatory cytokines and reactive oxygen species, which enhance hepatic inflammation and fibrosis (Rivera et al. 2008). CD36, which is a scavenger receptor, plays an important role of the NASH development (He et al. 2011). Hepatic resident macrophages express CD36, and take up oxidized low‐density lipoprotein (oxLDL), which contributes to accumulation of fat in the liver (Kunjathoor et al. 2002; Moore and Freeman 2006). Moreover, CD36 recognizes fatty acids such as palmitate and oleic acid, which activate intracellular signaling pathways that induce pro‐inflammatory cytokines and reactive oxygen species (Rahaman et al. 2006). Interestingly, CD36 knockout mice show lower hepatic fibrosis, and inflammation (Bieghs et al. 2010; Kennedy et al. 2011). These evidences indicate that CD36 signaling mediates NASH development via activation of inflammatory response.

Although feeding of high‐fat diet increased the expression of peroxisome proliferator‐activated receptor (PPAR)‐γ and nuclear factor erythroid‐2 related factor (Nrf)‐2 in the liver (Inoue et al. 2005; Huang et al. 2010), these transcription factors induce fatty liver disease *via* up‐regulation of CD36 expression. Interestingly, previous studies reported that PPAR‐γ and Nrf‐2 promote up‐regulation of CD36 expression in macrophages (Tontonoz et al. 1998; Ishii et al. 2004). Therefore, obesity‐induced up‐regulation of PPAR‐γ and Nrf‐2 might be an important factor for increasing CD36 expression in hepatic resident macrophages.

Exercise training is considered a crucial event leading to reduced inflammation in the liver and adipose tissue (Gleeson et al. 2011). We have shown that exercise training attenuates hepatic inflammation and fibrosis in obese mice on high‐fat, high‐fructose water diet (Kawanishi et al. 2012). Accordingly, it is possible that exercise training attenuates hepatic inflammation and fibrosis in liver tissue by suppressing CD36 expression in diet‐induced NASH model mice.

The purpose of the present study was to determine the effects of exercise training on CD36 expression in NASH model mice. Our hypothesis was that exercise training suppresses CD36 expression on hepatic macrophages by suppressing PPAR‐γ and Nrf‐2.

## Materials and Methods

### Animals and diets

Four‐week‐old male C57BL/6 mice were purchased from Kiwa Laboratory Animals (Wakayama, Japan). The experimental procedures followed the Guiding Principles for the Care and Use of Animals of the Waseda University Institutional Animal Care and Use Committee. The mice were randomly divided into four groups: normal diet (ND) and sedentary (*n* = 7), ND with exercise training (*n* = 5), high‐fat, high‐fructose water diet (HFF) and sedentary (*n* = 11), and HFF with exercise training (*n* = 11) groups. Mice of HFF group were fed a high‐fat diet (D12492; Research Diets, New Brunswick, NJ) and high‐fructose water contained 21% (wt/V) fructose (Sigma, St. Louis, MO). The high‐fat diet was composed of 60% fat, 20% protein, and 20% carbohydrate (of total calories). Mice of ND group were fed a standard normal diet. Normal diet was composed of 10% fat, 20% protein, and 70% carbohydrate (D12450B; Research Diets, New Brunswick, NJ) and fructose‐free water.

Exercise training was initiated when the mice were four weeks of age and continued for sixteen weeks. Mice were placed on a motorized treadmill for 60 min (during the light phase)/day, 5 days/week. The exercise speed was set at 15–20 m/min. Mice in the exercise group were not subjected to electric shock during treadmill running. The sedentary and exercise‐trained mice were dissected 3 days after the final training session under light anesthesia with isoflurane (Abbott, Tokyo, Japan). The livers were quickly removed, weighed, frozen in liquid nitrogen, and stored at −80°C until analysis. All mice were sacrificed by cervical dislocation under anesthesia.

### Real‐time quantitative polymerase chain reactions (PCR)

Total RNA was extracted from the liver using RNeasy Mini Kit (Qiagen, Valencia, CA). Total mRNA was reverse transcribed to cDNA using High Capacity cDNA Reverse Transcription Kit (Applied Biosystems). Total RNA was reverse transcribed to cDNA using the High Capacity cDNA Reverse Transcription Kit (Applied Biosystems). PCR was performed using the Fast 7500 real‐time PCR system (Applied Biosystems) and SYBR^®^ Green PCR Master Mix (Applied Biosystems). The conditions of reactions were as follows: initial 10 min denaturation step at 95°C, followed by 40 cycles of 95°C for 3 sec and at 60°C for 15 sec. Glyceraldehyde‐3‐phosphate dehydrogenase (GAPDH) was used as the housekeeping gene. The sequences of the primers are shown in Table 1.

**Table 1:** Primer sequences for real‐time RT‐PCR analysis

Gene	Forward	Reverse
GAPDH	TGAAGCAGGCATCTGAGGG	CGAAGGTGGAAGAGTGGGAG
CD36	CCGGGCCAACGTAGAAAACA	CCTCCAAACACAGCCAGGAC
PPARγ	CAAGAATACCAAAGTGCGATCAA	GAGCAGGGTCTTTTCAGAATAATAAG
Nrf2	CTCGCTGGAAAAAGAAGTGG	CCGTCCAGGAGTTCAGAGAG

### Western blot

Liver was homogenized (Polytron) in ice‐cold T‐PER Tissue Protein Extraction Reagent (Thermo Fisher Scientific) supplemented with Halt Protease Inhibitor Cocktail (Thermo Fisher Scientific). Homogenates were centrifuged at 12,000*g* for 15 min, and the supernatant removed. Protein concentrations of the liver lysates were subsequently determined using BCA Protein Assay Kit (Thermo Fisher Scientific). Liver lysates were solubilized in Laemmeli sample buffer (BioRad) and heated at 60°C for 30 min. Samples (30 μg protein) were subjected to 10% SDS‐polyacrylamide gel electrophoresis, and transferred to a PVDF membrane, blocked with PVDF Blocking Reagent (TOYOBO, Tokyo, Japan), and incubated with the primary antibody [1:2000 PPARγ (Cell Signaling Technology: CST), 1:2000 NRF2 (CST), and 1:2000 β‐actin (CST)], and finally with horseradish peroxide (HRP)‐linked secondary antibody [1:5000 dilution for anti‐rabbit IgG (CST)]. β‐actin was used as an internal control. Blotted samples were analyzed using ECL Advance Western Blotting Detection Kit (GE Healthcare Japan, Tokyo, Japan), and quantified by densitometry.

### Isolation of hepatic mononuclear cells and flow cytometry analysis

After removal of blood from liver, the liver was minced with scissors. Hanks’ Balanced Salt Solution (HBSS) containing 0.05% collagenase type 4 (Worthington, Lakewood, NJ) was added to the pieces of liver tissue. The mixture was shaken for 20 min at 37°C, and the digested tissue was centrifuged. The resultant pellet was filtered through a 70‐μm stainless steel mesh, and the digested tissue was centrifuged. The resultant pellet containing was suspended in 12 ml 33% Percoll (Sigma) solution, and a 15 min centrifugation step at 500*g* was performed. The resultant pellet containing the mononuclear cells was suspended in 5 mL Red Blood Cell Lysing buffer (Sigma) and filtered through a 40‐μm nylon mesh. The cells were washed twice with Stain Buffer (BD Pharmingen, Franklin Lakes, NJ), and the mononuclear cells were resuspended in Stain Buffer. The mononuclear cells (2.5 × 10^5^ cells) were incubated with Fc‐blocker (anti‐CD16/CD32) for 20 min followed by staining with PE‐Cy7‐CD11b (eBioscience, San Diego, CA), PE‐Cy5‐F4/80, PE CD36 for 20 min. Flow cytometry was performed using a Guava^®^ EasyCyte^TM^ 6HT and InCyte software (Millipore, Long Beach, CA). We validated flow cytometric identification of macrophages (CD11b^+^ F4/80^+^) and CD36 expression in macrophages. Hepatic resident mononuclear cells were gated according to side scatter and forward scatter plots. Hepatic resident macrophage populations were gated according to CD11b^+^ and F4/80^+^ on the side scatter and forward scatter plot. Geometric mean florescence intensity of the CD36 in macrophages (CD11b^+^ F4/80^+^) gated population was obtained to quantify cell surface expression on macrophages.

### Statistical analyses

All values are expressed as means ± SEM. Statistical analyses were performed using SPSS V17.0. For the comparison of all groups, a two‐way analysis of variance (ANOVA) was performed with diet (ND or HFF) and exercise (control or exercise training). If significant interactions were observed in any of these analyses, and comparisons with the Bonferroni correlated post hoc test were performed. The alpha level was set at *P* < 0.05.

## Results

**Figure 1:**
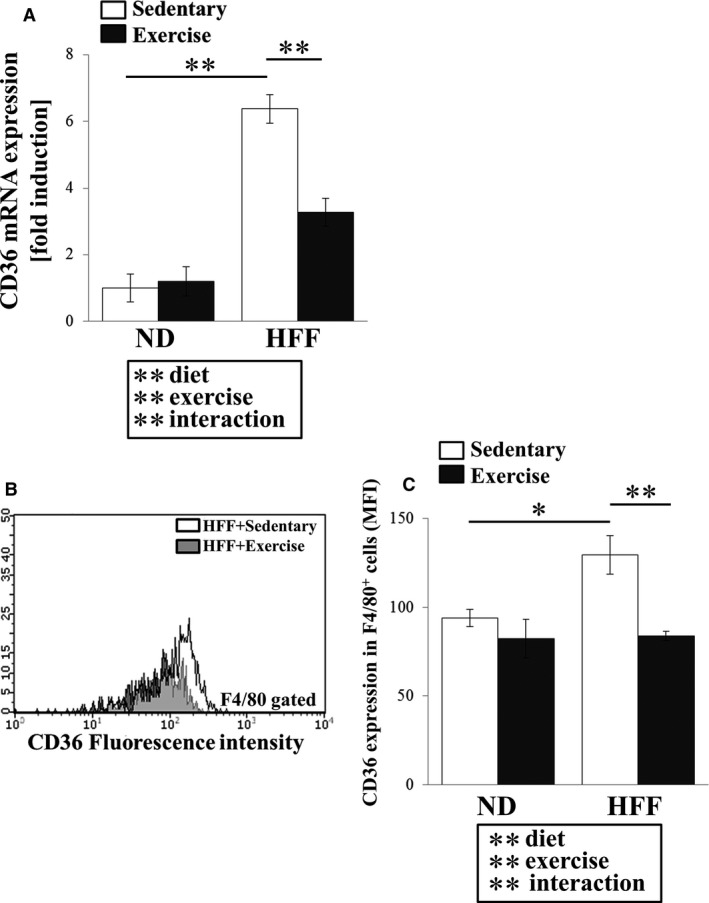
Effect of exercise training on CD36 expression in normal diet (ND)‐ and high‐fat and high‐fructose water (HFF)‐fed mice. CD36 mRNA expression in the liver (A). Flow cytometric histograms represent levels of CD36 (B) and geometric mean fluorescence intensity (MFI) expression (C) in hepatic macrophages (F4/80^+^ cells). Values represent means ± SEM. Analyses were performed using two‐way ANOVA for multiple comparisons (boxed text). **P <* *0.01, *P <* *0.05.

At the CD36 mRNA level, we found a significant diet x exercise interaction (*F*_1,30_ = 63.27, *P* < 0.01). However, we also found elevated CD36 mRNA in the HFF sedentary mice than in ND sedentary mice (*P* < 0.01), whereas the CD36 mRNA expression in HFF exercise mice was lower than in the HFF sedentary mice (*P* < 0.01, Fig. 1A). The changes by diet and exercise training in surface expression of CD36 in macrophages are shown in Figure 1B and C. Similar to CD36 mRNA expression in the liver, we also found a significant diet x exercise interaction with respect to CD36 expression by hepatic resident macrophages (*F*_1,28_ = 4.18, *P* < 0.05). *Post hoc* comparisons revealed that CD36 expression of hepatic resident macrophages was significantly higher in the HFF sedentary mice compared to the ND sedentary mice (*P* < 0.05), but this expression was significantly lower in HFF exercise mice than in HFF sedentary mice (*P* < 0.01, Fig. 1C).

**Figure 2:**
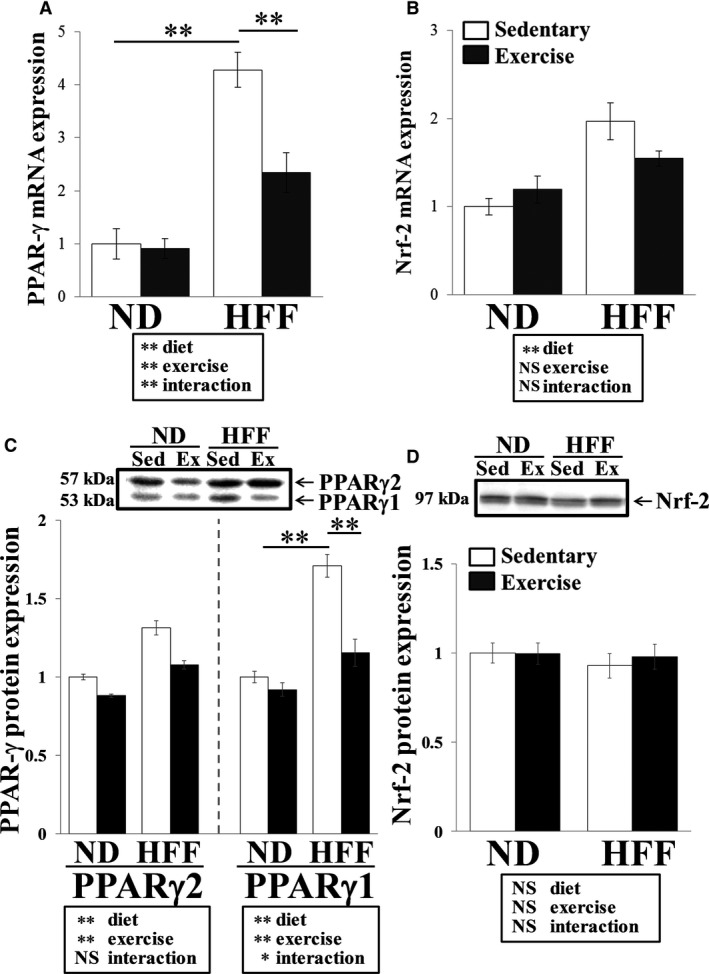
Effect of exercise training on PPAR‐γ and Nrf‐2 expression in ND‐ and HFF‐fed mice. PPAR‐γ (Α) and Nrf‐2 (B) mRNA expression in the liver. PPAR‐γ (C) and Nrf‐2 (D) protein expression in the liver. Values represent means ± SEM. Analyses were performed using two‐way ANOVA for multiple comparisons (boxed text). NS; not significant, **P <* *0.01, *P <* *0.05.

PPAR‐γ and Nrf‐2 are thought to play a crucial role in up‐regulation of CD36. PPAR‐γ mRNA levels in the liver varied as a significant diet x exercise interaction (*F*_1,30_ = 40.16, *P* < 0.01). *Post hoc* comparisons revealed that the PPAR‐γ mRNA level was significantly higher in HFF sedentary mice compared with ND sedentary mice (*P* < 0.01). However, PPAR‐γ mRNA level in HFF exercise mice was significantly lower than in HFF sedentary mice (*P* < 0.01, Fig. 2A). In contrast, Nrf‐2 mRNA level in the liver was significantly affected by diet (*F*_1,30_ = 15.25, *P* < 0.01), but a diet x exercise interaction was not observed (Fig. 2B). The changes in PPAR‐γ and Nrf‐2 protein expression levels are shown in Figure 2C and D. PPAR‐γ1 protein levels in the liver varied as a significant diet x exercise interaction (*F*_1,24_ = 5.908, *P* < 0.01). Similar to PPAR‐γ mRNA level, *post hoc* comparisons revealed that the PPAR‐γ1 protein level was significantly higher in HFF sedentary mice compared with ND sedentary mice (*P* < 0.01), but this level in HFF exercise mice was significantly lower than in HFF sedentary mice (*P* < 0.01, Fig. 2C). Although the PPAR‐γ2 protein level varied according to diet (*F*_1,24_ = 33.7, *P* < 0.01) and exercise (*F*_1,24_ = 11.2, *P* < 0.01), the interaction was not significantly different (Fig. 2C). In contrast, diet and/or exercise did not affect Nrf‐2 protein level (Fig. 2D).

**Figure 3:**
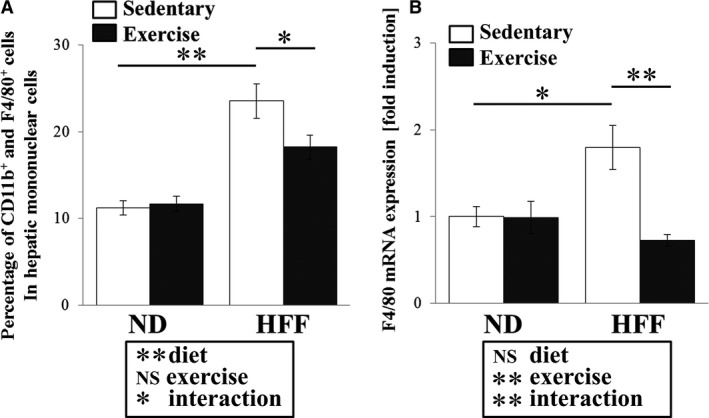
Effect of exercise training on hepatic macrophage infiltration in ND‐ and HFF‐fed mice. Populations of CD11b^+^ and F4/80^+^ cells in hepatic mononuclear cells were determined using flow cytometry (A). F4/80 mRNA expression in the liver (B). Values represent means ± SEM. Analyses were performed using two‐way ANOVA for multiple comparisons (boxed text). NS; not significant, **P <* *0.01, *P <* *0.05.

To determine the presence of macrophage in liver, we analyzed the percentage of hepatic resident macrophages (CD11b^+^ F4/80^+^ cells) in mononuclear cells and F4/80 mRNA expression in the liver. We found a significant diet x exercise interaction for population of CD11b^+^ F4/80^+^ cells (*F*_1,28_ = 4.38, *P* < 0.05). *Post hoc* comparisons revealed that this population of CD11b^+^ F4/80^+^ cells was significantly higher in the HFF sedentary mice than in the ND sedentary mice (*P* < 0.01), but this population was significantly lower in HFF exercise mice than in HFF sedentary mice (*P* < 0.05, Fig. 3A). We also observed a significant diet x exercise interaction for F4/80 mRNA level in the liver (*F*_1,30_ = 7.29, *P* < 0.05). *Post hoc* comparisons revealed that compared with ND sedentary mice, HFF sedentary mice had increased F4/80 mRNA levels in the liver (*P* < 0.05). However, this mRNA was decreased in HFF exercise mice compared with HFF sedentary mice (*P* < 0.01, Fig. 3B).

## Discussion

Scavenger receptor CD36 may play a role in the pathogenesis of NASH by modulating hepatic resident macrophages. CD36 recognizes fatty acids such as palmitate and oleic acid, which activate intracellular signaling pathways that induce pro‐inflammatory cytokines (Rahaman et al. 2006). Expression of CD36 is also elevated in the liver of high‐fat diet‐induced obese mice (Koonen et al. 2007). Interestingly, CD36 knockout mice showed both lower hepatic inflammation and fibrosis than wild‐type mice (Bieghs et al. 2010; Kennedy et al. 2011). A recent study showed that specific deletion of CD36 in bone marrow cells reduced hepatic inflammation and injury in high‐fat and high‐cholesterol diet‐fed mice (Bieghs et al. 2012). Therefore, scavenger receptor CD36 in macrophages may contribute to the development of hepatic inflammation and injury.

Although we have shown that exercise training attenuates hepatic inflammation and fibrosis in diet‐induced obese mice (Kawanishi et al. 2012), it remains unclear whether exercise training attenuates CD36 expression in the liver. CD36 is expressed strongly on the surface of monocytes taken from obese patients (Cipolletta et al. 2005). Butcher et al. (2008) reported that exercise training decreased CD36 and PPAR‐γ mRNA expression in leukocytes of healthy humans. In this study, we observed that exercise training decreased CD36 mRNA expression in the liver of obese mice. Although CD36 is mainly expressed in hepatic resident macrophages, exercise training also attenuated cell surface expression of CD36 in hepatic resident macrophages. In contrast, CD36 expression in blood monocytes was not changed by exercise training (data not shown). These results suggest that exercise training may possibly improve hepatic inflammation and fibrosis by reducing CD36 expression of hepatic resident macrophages in the NASH model mice.

We have reported in our previous published paper the effects of obesity by HFF administration and exercise training (Kawanishi et al. 2012). HFF administration induced the gain of body mass, adipose tissue mass, liver mass, and hepatic triglyceride accumulation. Importantly, exercise training decreased liver mass and hepatic triglyceride accumulation in HFF mice (Kawanishi et al. 2012). Since it was shown that CD36 regulated fat uptake in liver, we investigated whether changes of CD36 expression in liver were associated with development of hepatic steatosis. Interestingly, we observed that CD36 mRNA levels in liver were correlated with the concentration of hepatic triglyceride (*r* = 0.712, *P* < 0.01, data not shown). Therefore, the suppression of CD36 expression in liver by exercise training may play a key role in the reduction in hepatic steatosis. Importantly, we also reported that body mass and adipose tissue mass of NASH model were not affected by exercise training (Kawanishi et al. 2012). Therefore, our findings indicate that exercise training may directly attenuate CD36 expression in liver regardless of whether exercise results in weight loss.

PPAR‐γ and Nrf‐2 are known to be associated with the NASH development. Yamazaki et al. (2011) reported that PPAR‐γ‐knockout mice showed decreased mRNA expression of CD36 and TNF‐α than wild‐type mice. Moreover, Huang et al. (2010) also reported that Nrf2‐knockout mice exhibited reduced (1) hepatic triglyceride accumulation, (2) PPAR‐γ expression and (3) CD36 gene expression compared to the control mice following administration of HFF. Indeed, this study showed that exercise training both decreased CD36 expression of hepatic resident macrophages, and PPAR‐γ protein content and gene expression in the liver of obese mice. In contrast, Nrf‐2 expression in the liver was not affected by exercise training. Therefore, the exercise training‐induced reduction in CD36 expression in the liver might be regulated by the down‐regulation of PPAR‐γ. Although we observed that exercise training attenuates PPAR‐γ expression in liver of NASH model mice, the mechanisms by which exercise training affects PPAR‐γ expression remain unclear. Future studies should investigate the mechanisms through which exercise training regulates PPAR‐γ.

In addition, another possibility for the decrease in CD36 mRNA expression in the liver following exercise training may be due to a decreased infiltration of macrophages. Obese mice show greater number of macrophages in adipose tissue, and infiltrating macrophages demonstrated increased CD36 expression (Bassaganya‐Riera et al. 2009). On the other hand, infiltration of hepatic resident macrophages was enhanced by feeding of high‐fat diet and high‐fructose water (Karlmark et al. 2009; Kohli et al. 2011). In this study, exercise training attenuated hepatic resident CD11b^+^ and F4/80^+^ cell population in the hepatic mononuclear cells. Furthermore, F4/80 mRNA expression in the liver was decreased by exercise training. These results suggest that exercise training might possibly attenuate CD36 expression in the liver by reducing hepatic macrophage infiltration.

The limitation of this study was that the experiment did not examine the activity of transcription factor such as PPAR‐γ and Nrf2 in the hepatic resident macrophages. CD36 is regulated by intracellular nuclear receptors, but analysis for transcription factor activity of intracellular PPAR‐γ and Nrf2 was not carried out. Therefore, future study is necessary to investigate the effect of exercise training on hepatic macrophage intracellular expression of transcription factors.

Collectively, we considered that the reduction in CD36 in the liver by exercise training might be associated with suppression of PPAR‐*γ*.

## Conflict of Interest

None declared.

## Data Accessibility

## References

[phy213902-bib-0001] Bassaganya‐Riera, J. , S. Misyak , A. J. Guri , and R. Hontecillas . 2009 PPAR gamma is highly expressed in F4/80(hi) adipose tissue macrophages and dampens adipose‐tissue inflammation. Cell. Immunol. 258:138–146. 19423085 10.1016/j.cellimm.2009.04.003 PMC2706276

[phy213902-bib-0002] Bieghs, V. , K. Wouters , P. J. van Gorp , M. J. Gijbels , M. P. de Winther , C. J. Binder , et al. 2010 Role of scavenger receptor A and CD36 in diet‐induced nonalcoholic steatohepatitis in hyperlipidemic mice. Gastroenterology 138:2477–2486. 20206177 10.1053/j.gastro.2010.02.051 PMC3114629

[phy213902-bib-0003] Bieghs, V. , F. Verheyen , P. J. van Gorp , T. Hendrikx , K. Wouters , D. Lütjohann , et al. 2012 Internalization of modified lipids by CD36 and SR‐A leads to hepatic inflammation and lysosomal cholesterol storage in Kupffer cells. PLoS ONE 7:e34378. 22470565 10.1371/journal.pone.0034378 PMC3314620

[phy213902-bib-0004] Browning, J. D. , and J. D. Horton . 2004 Molecular mediators of hepatic steatosis and liver injury. J. Clin. Invest. 114:147–152. 15254578 10.1172/JCI22422 PMC449757

[phy213902-bib-0005] Butcher, L. R. , A. Thomas , K. Backx , A. Roberts , R. Webb , and K. Morris . 2008 Low‐intensity exercise exerts beneficial effects on plasma lipids via PPARgamma. Med. Sci. Sports Exerc. 40:1263–1270. 18580406 10.1249/MSS.0b013e31816c091d

[phy213902-bib-0006] Cipolletta, C. , K. E. Ryan , E. V. Hanna , and E. R. Trimble . 2005 Activation of peripheral blood CD14 + monocytes occurs in diabetes. Diabetes 54:2779–2786. 16123369 10.2337/diabetes.54.9.2779

[phy213902-bib-0007] Gleeson, M. , N. C. Bishop , D. J. Stensel , M. R. Lindley , S. S. Mastana , and M. A. Nimmo . 2011 The anti‐inflammatory effects of exercise: mechanisms and implications for the prevention and treatment of disease. Nat. Rev. Immunol. 11:607–615. 21818123 10.1038/nri3041

[phy213902-bib-0008] He, J. , J. H. Lee , M. Febbraio , and W. Xie . 2011 The emerging roles of fatty acid translocase/CD36 and the aryl hydrocarbon receptor in fatty liver disease. Exp. Biol. Med. 236:1116–1121. 10.1258/ebm.2011.011128 21885479

[phy213902-bib-0009] Huang, J. , I. Tabbi‐Anneni , V. Gunda , and L. Wang . 2010 Transcription factor Nrf2 regulates SHP and lipogenic gene expression in hepatic lipid metabolism. Am. J. Physiol. Gastrointest. Liver Physiol. 299:1211–1221. 10.1152/ajpgi.00322.2010 PMC3006243 20930048

[phy213902-bib-0010] Inoue, M. , T. Ohtake , W. Motomura , N. Takahashi , Y. Hosoki , S. Miyoshi , et al. 2005 Increased expression of PPARgamma in high fat diet‐induced liver steatosis in mice. Biochem. Biophys. Res. Commun. 336:215–222. 16125673 10.1016/j.bbrc.2005.08.070

[phy213902-bib-0011] Ishii, T. , K. Itoh , E. Ruiz , D. S. Leake , H. Unoki , M. Yamamoto , et al. 2004 Role of Nrf2 in the regulation of CD36 and stress protein expression in murine macrophages: activation by oxidatively modified LDL and 4‐hydroxynonenal. Circ. Res. 94:609–616. 14752028 10.1161/01.RES.0000119171.44657.45

[phy213902-bib-0012] Karlmark, K. R. , R. Weiskirchen , H. W. Zimmermann , N. Gassler , F. Ginhoux , C. Weber , et al. 2009 Hepatic recruitment of the inflammatory Gr1 + monocyte subset upon liver injury promotes hepatic fibrosis. Hepatology 50:261–274. 19554540 10.1002/hep.22950

[phy213902-bib-0013] Kawanishi, N. , H. Yano , T. Mizokami , M. Takahashi , E. Oyanagi , and K. Suzuki . 2012 Exercise training attenuates hepatic inflammation, fibrosis and macrophage infiltration during diet induced‐obesity in mice. Brain Behav. Immun. 26:931–941. 22554494 10.1016/j.bbi.2012.04.006

[phy213902-bib-0014] Kennedy, D. J. , S. Kuchibhotla , K. M. Westfall , R. L. Silverstein , R. E. Morton , and M. Febbraio . 2011 A CD36‐dependent pathway enhances macrophage and adipose tissue inflammation and impairs insulin signaling. Cardiovasc. Res. 89:604–613. 21088116 10.1093/cvr/cvq360 PMC3028977

[phy213902-bib-0015] Kohli, R. , M. Kirby , S. A. Xanthakos , S. Softic , A. E. Feldstein , V. Saxena , et al. 2011 High‐fructose, medium chain trans fat diet induces liver fibrosis and elevates plasma coenzyme Q9 in a novel murine model of obesity and nonalcoholic steatohepatitis. J. Nutr. Biochem. 22:543–553. 20607689 10.1002/hep.23797 PMC2932817

[phy213902-bib-0016] Koonen, D. P. , R. L. Jacobs , M. Febbraio , M. E. Young , C. L. Soltys , H. Ong , et al. 2007 Increased hepatic CD36 expression contributes to dyslipidemia associated with diet‐induced obesity. Diabetes 56:2863–2871. 17728375 10.2337/db07-0907

[phy213902-bib-0017] Kunjathoor, V. V. , M. Febbraio , E. A. Podrez , K. J. Moore , L. Andersson , S. Koehn , et al. 2002 Scavenger receptors class A‐I/II and CD36 are the principal receptors responsible for the uptake of modified low density lipoprotein leading to lipid loading in macrophages. J. Biol. Chem. 277:49982–49968. 12376530 10.1074/jbc.M209649200

[phy213902-bib-0018] Maher, J. J. , P. Leon , and J. C. Ryan . 2008 Beyond insulin resistance: innate immunity in nonalcoholic steatohepatitis. Hepatology 48:670–678. 18666225 10.1002/hep.22399 PMC3592568

[phy213902-bib-0019] Moore, K. J. , and M. W. Freeman . 2006 Scavenger receptors in atherosclerosis: beyond lipid uptake. Arterioscler. Thromb. Vasc. Biol. 26:1702–1711. 16728653 10.1161/01.ATV.0000229218.97976.43

[phy213902-bib-0020] Rahaman, S. O. , D. J. Lennon , M. Febbraio , E. A. Podrez , S. L. Hazen , and R. L. Silverstein . 2006 A CD36‐dependent signaling cascade is necessary for macrophage foam cell formation. Cell Metab. 4:211–221. 16950138 10.1016/j.cmet.2006.06.007 PMC1855263

[phy213902-bib-0021] Rivera, C. A. , P. Adegboyega , N. van Rooijen , A. Tagalicud , M. Allman , and M. Wallace . 2008 Toll‐like receptor‐4 signaling and Kupffer cells play pivotal roles in the pathogenesis of non‐alcoholic steatohepatitis. J. Hepatol. 47:571–579. 10.1016/j.jhep.2007.04.019 PMC2094119 17644211

[phy213902-bib-0022] Tontonoz, P. , L. Nagy , J. G. Alvarez , V. A. Thomazy , and R. M. Evans . 1998 PPARgamma promotes monocyte/macrophage differentiation and uptake of oxidized LDL. Cell 93:241–252. 9568716 10.1016/s0092-8674(00)81575-5

[phy213902-bib-0023] Yamazaki, T. , S. Shiraishi , K. Kishimoto , S. Miura , and O. Ezaki . 2011 An increase in liver PPAR*γ*2 is an initial event to induce fatty liver in response to a diet high in butter: PPAR*γ*2 knockdown improves fatty liver induced by high‐saturated fat. J. Nutr. Biochem. 22:543–553. 20801631 10.1016/j.jnutbio.2010.04.009

